# Perceived quality of care among people with type 2 diabetes mellitus in the north east region of peninsular Malaysia

**DOI:** 10.1186/s12889-021-10320-y

**Published:** 2021-02-02

**Authors:** Noorfariza Nordin, Suhaily Mohd Hairon, Najib Majdi Yaacob, Anees Abdul Hamid, Seoparjoo Azmel Mohd Isa, Norzaihan Hassan

**Affiliations:** 1 Department of Community Medicine, School of Medical Sciences, Health Campus, Universiti Sains Malaysia, 16150 Kubang Kerian, Kelantan Malaysia; 2 Unit of Biostatistics and Research Methodology, School of Medical Sciences, Health Campus, Universiti Sains Malaysia, 16150 Kubang Kerian, Kelantan Malaysia; 3 Primer Health Unit, Kelantan State Health Department, Aras 5, Wisma Persekutuan Kota Bharu, Jalan Bayam, 15590 Kota Bharu, Kelantan Malaysia; 4 Department of Pathology, School of Medical Sciences, Health Campus, Universiti Sains Malaysia, 16150 Kubang Kerian, Kelantan Malaysia; 5 Hospital Universiti Sains Malaysia, 16150 Kubang Kerian, Kelantan Malaysia; 6 Kota Bharu Health Clinic, Jalan Hospital, Bandar Kota Bharu, 15000 Kota Bharu, Kelantan Malaysia

**Keywords:** Perceived quality of care, PACIC, PACIC-M, Primary healthcare, Type 2 diabetes mellitus, Family doctor concept

## Abstract

**Background:**

People with type 2 diabetes mellitus (T2DM) are best managed by a chronic care model that is associated with enhanced quality of care and improved patient outcome. Assessing patients’ perceived quality of care is crucial in improving the healthcare delivery system. Hence, this study determined the perceived quality of care among people with T2DM and explored its associations with (i) sociodemographic and clinical characteristics and (ii) types of healthcare clinics to guide future planning.

**Methods:**

A cross-sectional study involving 20 primary healthcare clinics in the North East Region of Peninsular Malaysia and people with T2DM as the sampling unit was conducted from February to May 2019. The pro forma checklist, interview-guided Skala Kepuasan Interaksi Perubatan-11, and Patient Assessment of Chronic Illness Care (Malay version; PACIC-M) questionnaire were used for data collection. Univariate analysis and linear regression were used to determine the status of perceived quality of care and the factors associated with the perceived quality of care, respectively.

**Results:**

Overall, data from 772 participants were analyzed. The majority was from the Malay ethnic group (95.6%) with a mean (standard deviation [SD]) glycated hemoglobin A1c (HbA1c) level of 8.91% (2.30). The median (interquartile range [IQR]) of the number of medical officers available at each clinic was 6 (7), with Family Doctor Concept (FDC) clinics having a higher number of medical officers than non-FDC clinics (*p* = 0.001). The overall mean (SD) PACIC-M score was 2.65 (0.54) with no significant difference between scores of patients treated in the two clinic types (*p* = 0.806). Higher perceived quality of care was associated with lower number of medical officers (adjusted regression coefficient [Adj.β], − 0.021; *p*-value [*p*], 0.001), and greater doctor–patient interaction in all domains: distress relief (Adj.β, 0.033; *p*, < 0.001), rapport (Adj.β, 0.056; *p*, < 0.001), and interaction outcome (Adj.β, 0.022; *p*, 0.003).

**Conclusion:**

Although there was no significant difference found between clinic type, this study reflects that patients are comfortable when managed by the same doctor, which may support a better doctor-patient interaction. A larger specialized primary care workforce could improve diabetes care in Malaysia.

## Background

Type 2 diabetes mellitus (T2DM) is a prevalent and increasing trend in Malaysia, causing constant pressure to healthcare providers [1]. People with T2DM are best managed with evidence-based chronic care model (CCM) that is associated with enhanced quality of care and improved patient outcome through a coordinated multidisciplinary plan [2–5]. The CCM consists of six dimensions: healthcare organization, delivery system design, clinical information system, patient self-management support, decision support, and use of community resources [6]. The Malaysian primary healthcare is at an utmost effort to strengthen diabetes care in accordance with the CCM [6]. Hence, assessing the perceived quality of care received by patients is significant to reflect the effectiveness of CCM delivered by primary healthcare and improve the healthcare delivery process and hence outcome of healthcare [7–10]. The Patient Assessment of Chronic Illness Care (PACIC) is one of the valid measuring tools to assess whether the quality of care patients received is the same with the requirement in the CCM [5, 11].

Malaysia, which consists of 13 states and 3 federal territories, is located in Southeast Asia. The South China Sea divides Malaysia into Peninsular and East Malaysia. Kelantan is a state in the northeast of Peninsular Malaysia and has 10 districts with a population of 1.8 million people [12]; 49.6% of the Kelantan population resides in the urban areas, while the rest in the rural areas [12]. The majority of the population is from the Malay ethnic group (94.4%).

According to the Malaysian National Health and Morbidity Survey in 2015, 59.3% of patients diagnosed with T2DM in Malaysia were managed by primary healthcare clinics [13], public facilities meant to be used by all Malaysians. The remaining sought diabetes care at private clinics or private hospital outpatient clinics. The primary healthcare services in Malaysia have been strengthened through the initiative of the Family Doctor Concept (FDC) by the Ministry of Health of Malaysia in 2013 with the aim of achieving “One Family One Doctor” [4]. The FDC restructures the primary healthcare services, whereby patients and population are taken care of by a specific primary healthcare team (PHCT) according to zone [4]. Subsequently, the Kelantan State Health Department has started to implement the FDC since 2015, and the number of healthcare clinics implementing it has been increasing by years. In 2017, there were 69 primary healthcare clinics throughout Kelantan, including 13 FDC and 56 non-FDC clinics. The practice of “One Family One Doctor” in primary healthcare will enhance the quality of care among patients with chronic illnesses, such as T2DM [4, 14]. Central to the principle of primary healthcare is the doctor–patient relationship by which the interaction occurs with one another, and fostering and maintaining such relationship in a complex, integrated primary healthcare delivery system is indeed challenging for healthcare clinicians and policymakers [15]. Nonetheless, the doctor–patient interaction can be used to predict the patient’s adherence to treatment [16–18], as satisfied patients are more likely to engage with diabetes care [19]. A cross-sectional study conducted in Leeds, United Kingdom, found a correlation between overall satisfaction and glycated hemoglobin A1c (HbA1c) level among the study participants, whereby higher satisfaction score correlated with lower HbA1c level [17].

The current practice requires a family medicine specialist (FMS) to deliver diabetes care in both FDC and non-FDC clinics. However, the number of FMS is limited, and the sharing of expertise is needed [20]. Hence, diabetes cares heavily relies on the availability of medical officers who do not hold a postgraduate qualification in primary care [20]. In both clinic settings, people with T2DM are seen together with those presenting with acute minor ailments, and medical officers need to equip themselves with comprehensive medical knowledge for both acute and chronic illnesses. The major differences are the availability of equipment (e.g., fundus camera and X-ray modality) and specialty services (e.g., FMS, radiology, laboratory, nutrition and diet, physiotherapy, and occupational therapy) that are more available at FDC clinics than at non-FDC clinics [21]. Hence, equipment and specialty services are shared between FDC and non-FDC clinics in the same district [21].

People with T2DM require comprehensive management according to the CCM, good doctor–patient relationship, and higher perceived quality of care to improve diabetes outcome. Furthermore, the implementation of FDC in Malaysia was expected to increase the satisfaction of doctor–patient interaction and improve the quality of the CCM for diabetes care by providing the “One Family One Doctor” concept. A previous study describing the difference in doctor–patient interaction satisfaction between people with T2DM who visited FDC and non-FDC clinics and determining the association between FDC clinic and doctor–patient interaction satisfaction toward glycemic control was performed and published [21]. However, it did not explore the aspect on perceived quality of care and whether doctor–patient interaction satisfaction influenced the perceived quality of care. Hence, this study aimed to determine the perceived quality of care received by people with T2DM and explore its associations with (i) sociodemographic and clinical characteristics and (ii) types of healthcare clinics to guide future planning.

## Methods

### Study design and setting

A cross-sectional study involving 20 primary healthcare clinics, organized into FDC (*n* = 10) and non-FDC (*n* = 10), throughout all 10 districts in Kelantan was conducted from February to May 2019. The profile of the selected 20 primary healthcare clinics was presented in another publication [21]. FDC clinics had more human and technical resources than non-FDC clinics. There were differences between FDC and non-FDC clinics in terms of the average number of people with T2DM who visited clinics per day (*p* = 0.018); number of medical officers (*p* = 0.005), pharmacists (*p* = 0.001), and physiotherapists (*p* < 0.001); and availability of a fundus camera (*p* = 0.003) and X-ray machine (*p* = 0.005) at the clinics [21]. The median (interquartile range [IQR]) of the number of medical officers available at each clinic was 6 [7], with FDC clinics having a higher number of medical officers (8.5 [4]) than non-FDC clinics (2 [3]) (*p* = 0.001) [21]. The significant differences in the aforementioned variables were controlled in multivariate analysis.

### Study population

People with T2DM who visited FDC and non-FDC clinics in each district of Kelantan from February to May 2019 and fulfilled the study criteria were included into the study, as well as those who were 18 years old and above and able to understand the Malay language and received follow-up at current health clinics at least twice within the last 1-year duration (2018–2019). Pregnant women with preexisting T2DM were excluded as they received a different set of clinical management, as well as those people with T2DM from outside the health clinics’ operational area (as confirmed by the diabetes educator at the designated clinics).

### Sampling method

**Fig. 1:**
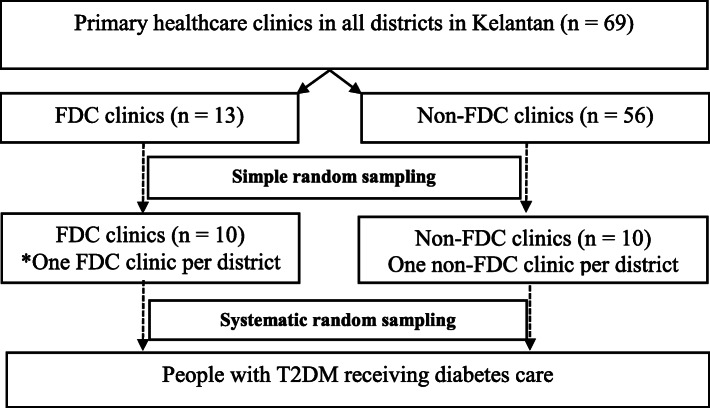
Sampling method. FDC, Family Doctor Concept; T2DM, type 2 diabetes mellitus

Multistage sampling technique was used (Fig. 1). Overall, 13 FDC and 56 non-FDC clinics were included for sampling into the study. Simple random sampling was conducted to select 1 FDC and 1 non-FDC clinic from each district to obtain a total of 10 FDC and 10 non-FDC clinics into the study. Systematic random sampling was performed on every data collection day at the clinics to select participants who fulfilled the study criteria.

### Participant recruitment

During the visit for data collection, each participant was identified with assistance from the diabetes educator at the designated healthcare clinics. The study was verbally explained to those who fulfilled the study criteria, and an information sheet was given. Written consent was obtained upon agreement to participate in the study.

### Study tools and data collection

This study used primary and secondary data collection techniques to gather the required information. Each clinic was visited for 3 days to recruit an adequate number of participants. People with T2DM were interviewed by a trained researcher using the pro forma checklist, Skala Kepuasan Interaksi Perubatan-11 (SKIP-11), and Patient Assessment of Chronic Illness Care (Malay version; PACIC-M) questionnaire. The pro forma checklist was used to collect information regarding age, sex, ethnicity, and marital and employment status during the structured interview. Meanwhile, data regarding diabetes mellitus duration, number of diabetes complications, and latest HbA1c reading were extracted from the patient’s diabetes record book. The complications of diabetes included retinopathy, nephropathy, erectile dysfunction, ischemic heart disease, cerebrovascular disease, diabetic foot ulcer, and/or amputation. The minimum and maximum numbers of complications were 0 and ≥ 4, respectively. Information regarding the average number of people with T2DM who visited the clinics per day and number of medical officers and diabetes educators were gathered from the Primary Care Unit, Kelantan State Health Department. The number of medical officers was the number of available medical officers (excluding FMS) working in a particular clinic and did not reflect the actual number of medical officers seen by a patient. Meanwhile, the number of diabetes educators was obtained as they play a significant role in diabetes education.

The SKIP-11 questionnaire was used to measure the satisfaction of doctor–patient interaction. It originated from the Medical Interview Satisfaction Scale-21 in the English language and was translated and validated to the Malay version by Abd Aziz et al. (2013) with good internal consistency value (Cronbach’s alpha = 0.669) [22]. It consisted of 11 items in 3 domains, namely, distress relief, rapport, and interaction outcome. The “distress relief” domain, which consists of four items, measured information provision by the doctor; the “rapport” domain, constructed by four items, measured the patient’s confidence in the doctor; and the “interaction outcome” domain, which consists of three items, reflected the doctor’s communication skills and adherence intent [19, 22]. Each item was scored on a 5-point Likert scale: for positively worded items, scores 1 and 5 were for “strongly disagree” and “strongly agree,” respectively, whereas scores were reversed for negatively worded items. For the overall SKIP-11 score, the minimum and maximum total scores were 11 and 55, respectively. For the domains, “distress relief” and “rapport” had a minimum score of 4 and maximum score of 20 each, and “interaction outcome” had minimum and maximum scores of 3 and 15, respectively [21].

Meanwhile, the PACIC-M questionnaire was used to measure the perceived quality of care received by people with T2DM. The original 20-item PACIC questionnaire was developed by Russell E Glasgow et al. (2005) in the English language and was translated to the Malay language and validated by Abdul Razak et al. (2018) with internal consistency value (Cronbach’s alpha = 0.94), hence named as the PACIC-M [6]. Data were collected with guidance from an interviewer, and patients needed to score their perceived quality of care received over the last 6 months. The questionnaire consisted of 19 items in 3 domains, as experienced by patients: (1) patient activation and decision support, (2) goal-setting and problem-solving, and (3) follow-up and coordination. Each item was scored on a 5-point scale, with 1 for “almost never” and 5 for “almost always” [6]. The patient activation and decision support domain had minimum and maximum scores of 5 and 25; goal-setting and problem-solving, 10 and 50; and follow-up and coordination, 4 and 20, respectively. The overall PACIC-M score was measured by averaging across all 19 items, and each domain was scored by averaging the score of items answered within each domain [6]. Higher scores indicated higher perceived quality of diabetes care.

### Sample size calculation

The number of healthcare clinics was set by the researcher at 20 healthcare clinics, with as low as 10 as the minimum required number of clusters in a hierarchical model [23]. The sample size was calculated for each variable of associated factors for perceived quality of care among people with T2DM using a sample size calculator for a comparison of two independent means [24]. The estimated sample for each group was 354 using the standard deviation (SD) of the overall PACIC score of 0.9 [25], detectable difference of 0.2, 5% type 1 error, 80% power, and additional 20% missing data. Therefore, the total sample size required was 796 people with T2DM. Simple random sampling for participant recruitment from all people with T2DM in Kelantan who fulfilled the study criteria was performed.

### Statistical analysis

Data were entered and analyzed using SPSS ver.24 and were explored to check for missing value and normality distribution. Data were presented as mean with SD for numerical variables and number (n) with percentage (%) for categorical variables. The independent sample t-test was used to determine the differences in the overall PACIC-M score and its domains between people with T2DM who visited FDC and non-FDC clinics. Simple and multiple linear regression models were used to identify factors associated with the overall PACIC-M score. Independent variables included age, sex, ethnicity, marital and employment status, diabetes mellitus duration, HbA1c, number of diabetes complications, health clinic type, average number of people with T2DM who visited the clinic per day, number of medical officers and diabetes educators, and doctor–patient interaction score. The outcome variable was the overall PACIC-M score. The variable of the overall SKIP-11 score was excluded from the final model, and only its domains were included to avoid multicollinearity. Stepwise, backward and forward methods were used to obtain the parsimonious model. Possible two-way interactions were checked. The final model was presented as an adjusted regression coefficient (Adj.β) with its 95% confidence interval (95% CI) and *p*-value. A two-tailed p-value of less than 0.05 was considered statistically significant.

### Results

**Fig. 2:**
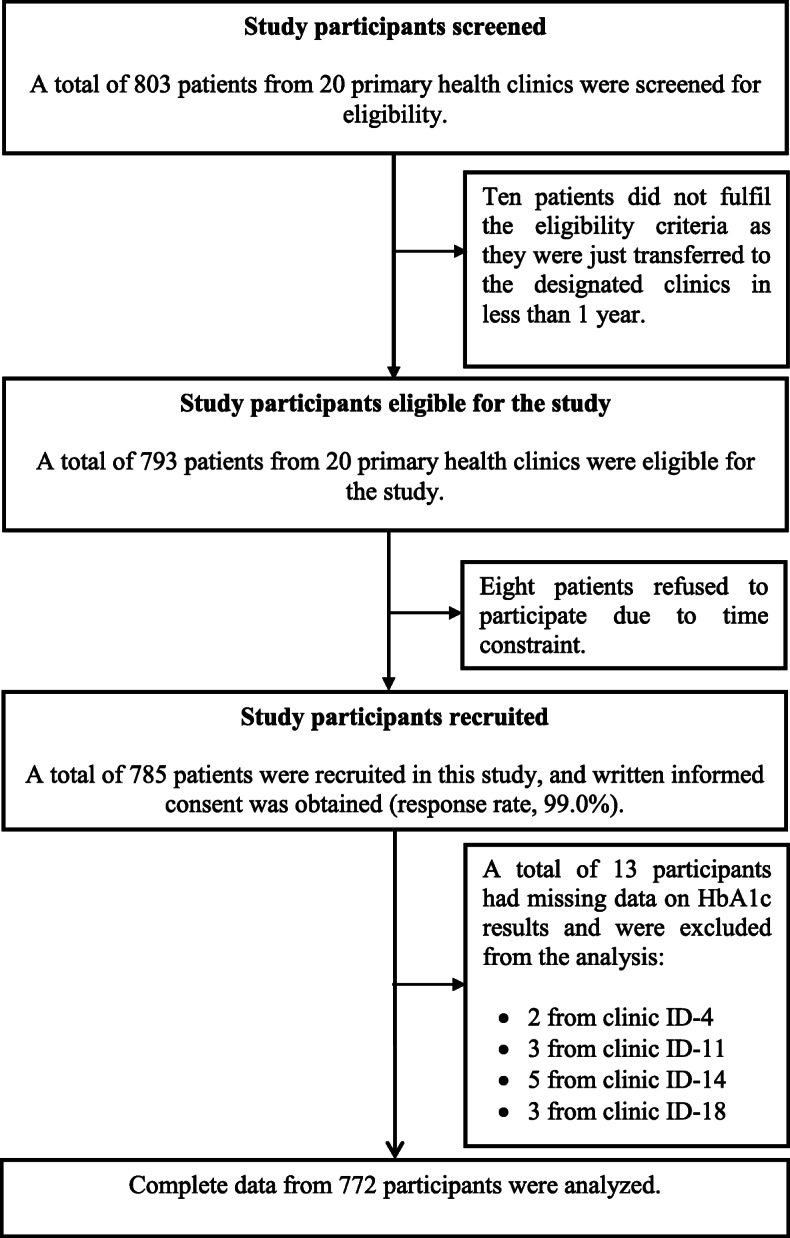
Flowchart of study participants

The flowchart of the study participant is shown in Fig. 2 (response rate, 99.0%). Complete data from 772 respondents with T2DM who participated in this study (434 from FDC and 338 from non-FDC clinics) were analyzed. The detailed profile of people with T2DM was also presented in another publication [21], and there were no differences between people with T2DM who visited both types of healthcare clinics, except for ethnicity (*p* < 0.001) [21] (Table 1). There were differences in the overall SKIP-11 score (*p* = 0.015) and distress relief domain (*p* = 0.008) between people who visited FDC and non-FDC clinics (Table 1).

**Table 1:** Profile of T2DM patients attended primary health clinics in Kelantan (*n* = 772)

Variables	Total (*n* = 772)		T2DM visited FDC clinics (***n*** = 434)		T2DM visited non-FDC clinics (***n*** = 338)		***p***-value
	n	(%)	n	(%)	n	(%)	
**Age** ^a^	60.57	(10.04)	60.41	(9.79)	60.77	(10.37)	0.609^e^
**Sex**							
Male	245	(31.7)	141	(32.5)	104	(30.8)	0.666^c^
Female	527	(68.3)	293	(67.5)	234	(69.2)	
**Ethnic**							
Malay	738	(95.6)	405	(93.3)	333	(98.5)	**< 0.001**^c^
Non-Malay	34	(4.4)	29	(6.7)	5	(1.5)	
**Marital status**							
Married	562	(72.8)	316	(72.8)	246	(72.8)	0.993^c^
Single/divorce	210	(27.2)	118	(27.2)	92	(27.2)	
**Education level**							
Lower education	737	(95.5)	411	(94.7)	326	(96.4)	0.246^c^
Higher education	35	(4.5)	23	(5.3)	12	(3.6)	
**Employment status**							
Employed	206	(26.7)	122	(28.1)	84	(24.9)	0.641^c^
Unemployed	566	(73.3)	312	(71.9)	254	(75.1)	
**Duration of diabetes (years)**^b^	6.40	(6.81)	6.39	(6.94)	6.43	(6.60)	0.751^d^
**Number of diabetic complications**	0	(1)	0	(1)	0	(1)	0.935^d^
**Latest HbA1c**^a^	8.91	(2.30)	8.77	(2.23)	9.10	(2.37)	**0.046**^e^
**Average T2DM patients per day**^a^	24.6	(11.9)	32.1	(9.41)	17.0	(9.12)	**< 0.002**^e^
**Number of medical officers**^b^	6	(7)	8.5	(4)	2	(3)	**0.001**^**d**^
**Number of diabetes educators**^b^	1	(1)	1.5	(1)	1	(0)	0.055^d^
**Overall doctor-patient interaction satisfaction score (SKIP-11)**^a^	43.16	(5.93)	43.62	(5.95)	42.58	(5.86)	**0.015**^e^
***SKIP-11 domain score***^a^							
**Distress relief**	15.79	(2.64)	15.94	(2.52)	15.59	(2.77)	**0.008**^**e**^
**Rapport**	16.27	(2.54)	16.37	(2.43)	16.15	(2.66)	0.188^e^
**Interaction outcome**	11.10	(2.57)	11.23	(2.69)	10.94	(2.42)	0.117^e^

^a^mean(SD), ^b^median (IQR), ^c^ Chi-square statistic, ^d^Mann-Whitney U-test, ^e^Independent t-test, *FDC* Family Doctor Concept

### Perceived quality of care among people with T2DM

The PACIC-M item “satisfy that my care was well managed” (Q4) achieved the highest mean score (SD) for both primary healthcare clinics (FDC, 4.15 [0.85]; non-FDC, 4.16 [0.79]), while the PACIC-M item “being contacted after visit to clinics” (Q15) had the lowest mean score in FDC (1.29 [0.67]) clinics (non-FDC, 1.35 [0.72]).

All PACIC-M domains showed no significant difference between patients who received treatment at FDC clinics and those at non-FDC clinics. The highest average score was for the “patient activation and decision support” domain (FDC, 3.39 [0.68]; non-FDC, 3.39 [0.74]), and the lowest was for the “follow-up and coordination” domain (FDC, 2.11 [0.86]; non-FDC, 2.02 [0.84]).

There was no difference in the overall PACIC-M score between both groups with the overall mean (SD) PACIC-M score 2.65 (0.52) in the FDC group and 2.66 (0.57) in the non-FDC group (*p* = 0.806) (Table 2).

**Table 2:** Comparison of overall PACIC-M score and domains among people with T2DM treated in primary health care clinics in Kelantan (*n* = 772)

PACIC-M	Mean (SD)			95% CI	***p***-value^a^
	Total (*n* = 772)	People with T2DM visited FDC clinics (*n* = 434)	People with T2DM visited non-FDC clinics (*n* = 338)		
Patient activation and decision support	3.40 (0.71)	3.39 (0.68)	3.39 (0.74)	(−0.10, 0.10)	0.996
Goal setting and problem solving	2.52 (0.62)	2.49 (0.61)	2.55 (0.65)	(−0.04, 0.14)	0.229
Follow-up/coordination	2.07 (0.85)	2.11 (0.86)	2.02 (0.84)	(−0.21, 0.03)	0.144
**Overall PACIC-M score**	**2.65 (0.54)**	**2.65 (0.52)**	**2.66 (0.57)**	**(−0.07, 0.09)**	**0.806**

^a^independent t-test, *FDC* Family Doctor Concept

### Perceived quality of care and its associated factors

The age and HbA1c level of people with T2DM, number of medical officers, and domains of doctor–patient interaction satisfaction (i.e., distress relief, rapport, and interaction outcome) were found to be significant factors associated with the overall PACIC-M score.

The increasing age of people with T2DM and higher number of medical officers were associated with lower perceived quality of care. Meanwhile, a 1% increase in the HbA1c level and one score higher for each SKIP-11 domain were associated with higher perceived quality of care. The final model is shown in Table 3.

**Table 3:** Factors associated with overall PACIC-M score among people with T2DM treated in primary health care clinics in Kelantan (*n* = 772)

Variables	n (%)	Overall PACIC-M score	Simple Linear Regression		Multiple Linear Regression^a^		
		Mean (SD)	Crude b (95% CI)	***p***-value	Adj. b (95% CI)	t-stat	***p***-value
**Age**			−0.011 (− 0.02, − 0.01)	**< 0.001**	− 0.008 (− 0.012, − 0.005)	−4.59	**< 0.001**
**Age**							
< 60 years old	338 (43.8)	2.73 (0.57)	Ref				
≥ 60 years old	434 (56.2)	2.59 (0.51)	−0.133 (− 0.210, − 0.057)	**0.001**			
**Sex**							
Male	245 (31.7)	2.62 (0.54)	Ref				
Female	527 (68.3)	2.67 (0.54)	0.045 (−0.037, 0.128)	0.279			
**Race**							
Non-Malay	34 (4.4)	2.49 (0.57)	Ref				
Malay	738 (95.6)	2.66 (0.54)	0.171 (−0.015, 0.358)	0.071			
**Marital status**							
Single/widowed	210 (27.2)	2.61 (0.50)	Ref				
Married	562 (72.8)	2.67 (0.56)	0.051 (−0.035, 0.137)	0.242			
**Employment status**							
Unemployed	566 (73.3)	2.65 (0.52)	Ref				
Employed	206 (26.7)	2.65 (0.59)	−0.007 (− 0.093,0.080)	0.878			
**Duration of DM, years**			0.006 (−0.002, 0.014)	0.143			
**Number of diabetic complications**			−0.018 (− 0.070, 0.034)	0.506			
**HbA1c**			0.020 (0.003, 0.036)	**0.019**	0.025 (0.010, 0.040)	3.24	**0.001**
**HbA1c**							
≤ 6.5%	126 (16.3)	2.51 (0.53)	Ref				
> 6.5%	646 (83.7)	2.68 (0.54)	0.169 (0.066, 0.272)	**0.001**			
**Clinic type**							
Non-FDC	10 (50.0)	2.66 (0.57)	Ref				
FDC	10 (50.0)	2.65 (0.52)	−0.008 (− 0.085, 0.069)	0.844			
**Average number of people with T2DM visited clinic per day**			0.000 (−0.003, 0.004)	0.834			
**Number of medical officers**			−0.022 (− 0.034, − 0.009)	**0.001**	− 0.021 (− 0.031, − 0.010)	−3.68	**0.001**
**Number of diabetic educators**			−0.035 (− 0.080, 0.010)	0.127			
**SKIP-11 domain score**							
Distress relief			0.068 (0.054, 0.081)	**< 0.001**	0.033 (0.018, 0.048)	4.29	**< 0.001**
Rapport			0.084 (0.070, 0.097)	**< 0.001**	0.056 (0.039, 0.072)	6.76	**< 0.001**
Interaction outcome			0.039 (0.024, 0.054)	**< 0.001**	0.022 (0.007, 0.036)	2.93	**0.003**

^a^Constant 1.382; Adjusted R^2^ = 23.1%; Stepwise, backward and forward multiple linear regression method applied; model assumptions were fulfilled; no multicollinearity and no interaction detected

*FDC* Family Doctor Concept

## Discussion

The current study observed that people with T2DM perceived equal quality of care in both types of healthcare clinics. Interestingly, the lower number of medical officers and higher satisfaction of doctor–patient interaction were associated with higher perceived quality of care. Nonetheless, people aged less than 60 years and those with higher HbA1c levels were also associated with higher PACIC scores.

The overall PACIC-M score in this study was higher than in a local study conducted by Lim et al. (2019) [26]. This could be due to the different items in the PACIC questionnaire used between the two studies, whereby Lim et al. (2019) used 11 items, while our study used 19 items. In addition, the relatively higher overall PACIC-M score in our study population may be due to the differences in ethnicity as our study had 96% Malay respondents and that by Lim et al. (2019) involved 70% Malay and 21% Chinese respondents. The Malay ethnic group tended to give a higher score than the Chinese [26]. This may be due to the differences in expectations toward healthcare delivery between the various ethnic groups in Malaysia.

This study showed that there was no difference in the mean PACIC-M score between the people who visited FDC and non-FDC clinics. The general improvements in healthcare delivery and continuous training in diabetes care conducted by the Kelantan State Health Department could contribute to the similar perceived quality of care in both settings. A systematic review and meta-analysis of 34 studies by Arditi et al. (2018) was also unable to prove that integrated care could produce a higher PACIC score. Although FDC clinics are expected to perform better comprehensive, continuous, and coordinated care on diabetes management, this study observed that non-FDC clinics are also able to deliver equal diabetes care with shared resources. Other clinic factors could be associated with a higher PACIC score; however, most of the clinic factors were not significant in this study.

This study found that the highest PACIC-M score was given to the “patient activation and decision support” domain, and there was no difference between both types of clinics. The finding was comparable to a study conducted by Ku and Kegels (2014) in the Philippines, which reported the highest and similar score in the domain, and there was no difference between a facility with a specialist and that managed by a regular doctor. The similar score between both types of clinics reflected that every doctor was able to activate their patients during consultation even though an FMS was better trained for specific counseling skills to empower patients with knowledge and skills [20, 27]. This finding could also be explained by the fact that there are insufficient FMS to deliver care in FDC clinics, as only one FMS is available per FDC clinic. Each FDC clinic is responsible to care for 3000–15,000 people in their catchment area [4], giving the ratio of FMS to patients per FDC clinic of 1:3000–15,000 people in our study. According to Ramli et al. (2019), at least 16,000 FMS are needed to care for a population of 32 million people in Malaysia. Internationally, a ratio of FMS to patients of 1:2000 has been recommended to be at par with developed countries, with currently only 400 FMS serving in primary healthcare in Malaysia [20] at a ratio of 1:80,000. To date, only 29 FMS are serving a population of 1.8 million people in Kelantan, and this is far from international standard.

However, a local study by Lim et al. (2019) reported a contradicting finding whereby the domain “patient activation and decision support” was reported as lowest scored [26]. The difference could be attributed to the differences in the items used by both studies as the study by Lim et al. (2019) used three items, whereas ours used five for patient activation and decision support, hence the difference in the PACIC scoring. Moreover, the difference in the patient activation score could be due to geographical and ethnicity factors. A study by Lim et al. (2019) showed that other ethnicities, especially Chinese, tended to give a lower score, and our study participants were mostly from the Malay ethnic group, who gave a higher score on the patient activation domain. This may be because the Malay ethnic group is easily satisfied with the care provided and has lower expectation toward healthcare delivery.

The study participants rated a lower PACIC-M score for “goal-setting and problem-solving,” which was indifferent between both types of clinics. A study in Taiwan reported contradicting findings with a higher score of the domain, and there was a difference between two different types of health facilities [28]. In this study, the facilities with and without the pay for performance (P4P) program were compared, whereby patients who received treatment under the program rated a much higher score than those who were not under the program (*p* < 0.001). This P4P program offers payment to a physician for achieving the clinical targets set by the health authority, but this is not present in Malaysian healthcare systems. People with diabetes treated in either FDC or non-FDC clinics only pay RM 1.00 and probably have lower expectation of the quality of care, considering the minimal payment required for care. In addition, the physician does not receive extra incentives for conducting more laboratory nor clinical examinations to their patients, as well as when patients are able to achieve the targeted glycemic control. All laboratory and clinical examinations and medications needed by people with diabetes are highly subsidized by the Malaysian government, resulting in an equal chance of testing and treatment for all diabetic patients, regardless of the type of primary healthcare clinics.

Additionally, to achieve individuals’ targeted diabetic goals, healthcare teams in both FDC and non-FDC clinics received adequate training to integrate the diabetes management with other specialties such as pharmacists, physiotherapists, dieticians, and nutritionists. Although non-FDC clinics have limited resident specialties, patients with diabetes who require further management by other specialties will be identified and given an appointment to do so. In contrast, healthcare providers enrolled under the P4P program in Taiwan were highly motivated to perform laboratory and other clinical examinations and obtained extra incentives once patients had better process measurement (e.g., more frequent HbA1c testing) and outcome (e.g., improved glycemic control) (Chiu et al., 2016), which are not applicable in the Malaysian context.

This study also found that “follow-up and coordination” was the least-perceived PACIC-M domain, and this was in line with studies conducted in the Philippines [27] and Texas [29]. The reason for the low perception of follow-up and coordination may be due to less referral made to other specialties, and the PHCT did not perform active phone calls nor practice home visits for patients with chronic illness whenever they missed an appointment. This may be due to the limited human resources to perform active phone calls as 14 (70%) of the health clinics in this study only have one diabetes educator [21]. A study showed that home visits by known healthcare workers would produce better perception in follow-up and coordination [27]. In addition, this current study found no difference in the “follow-up and coordination” domain between FDC and non-FDC clinics. The gold standard in implementing FDC clinics is to have “One Family One Doctor,” and the recommended ratio is 1 FMS for every population of 2000 people [20] to improve the quality and continuity of care. Currently, at least one FMS is available in each FDC clinic that is responsible for a population of 3000–15,000 people [4]. The number of FMS in implementing FDC is insufficient in the first place, and the management of patients is highly dependent on medical officers, by which not all of them are specifically trained to provide primary healthcare services. Ideally, patients should be seen by the same doctor and PHCT during their follow-up appointment. However, due to the high turnover of doctors in health clinics by approximately 2 years, achieving the goal is a challenge, and sometimes, patients need to be seen by another doctor from other teams [4, 30]. The challenge of high turnover rate among doctors is an issue in non-FDC clinics too, and this may contribute to the absence of differences in the perception of “follow-up and coordination” between the two types of health clinics in Kelantan. The continuity of care for patients with T2DM in both types of health clinics needs to rely on FMS, diabetes educators, nurses, and medical assistants who frequently remain in the same health clinic for several years [30]. Furthermore, follow-up was provided according to the patient’s conditions and glycemic control, whereby patients with poor glycemic control would have frequent follow-ups and those with good glycemic control would have fewer follow-ups, irrespective of which settings they received the treatment.

An increase in the number of medical officers was associated with reduced PACIC-M score, and it reflected that patients felt more comfortable when they were managed by a similar doctor as they can communicate without repetitively mentioning their social and personal history. Patients highly value the continuity of care delivered by a familiar family doctor who they can develop rapport and long-term doctor–patient relationship. The healthcare provider’s knowledge of patients’ social aspect (e.g., living condition) and ability to explain matters in a simpler way to be understood by patients may increase the PACIC score [26]. Nevertheless, the current study observed that the higher SKIP-11 domain scores for “distress relief,” “rapport,” and “interaction outcome” were associated with higher PACIC-M scores. These findings further support the FDC of having “One Family One Doctor.” However, the implementation of FDC needs to be improved in the Malaysian primary healthcare clinics, especially for chronic long-term conditions such as diabetes. The first step forward is truly understanding what FDC means and what would the long-term goals for this initiative be, that is, to improve the quality, continuity, and coordination of care delivered by qualified family physicians. However, this concept will be unable to rectify the problems immediately, as the number of FMS needed to care for a defined population at an acceptable ratio at present is insufficient. One of the crucial measures to ensure the successful implementation of FDC is to increase the number of FMS in the public primary healthcare clinics in Malaysia. Although the ratio of all doctors (irrespective of specialty) to the population in Kelantan (1:812) seems to be adequate as reported by the Malaysian Health Facts (2018), not all doctors are specifically trained to provide primary healthcare services [31]. Ideally, 900 FMS are needed to care for a population of 1.8 million people in Kelantan. The adequacy in the number of FMS is significant as they are being trained to provide comprehensive, continuous, coordinated, and high-quality long-term care for patients with chronic conditions such as diabetes. The 4-year specialist training for FMS already includes problem-solving skills, decision support using clinical practice guidelines, shared decision-making, and goal-setting skills to increase patient activation, in line with the CCM [20].

Nonetheless, increasing age was associated with a reduced PACIC-M score, and this finding was similar to that of another study [26]. Older people may have difficulty in expressing their perception during interview, and the interviewer may also need assistance from the caretakers to exactly understand what the patients are trying to imply. In addition, older people may perceive lower quality of care because they often used traditional remedies and had lower expectation to medical care.

Nevertheless, this current study also observed that an increased HbA1c level was associated with an increased PACIC-M score. People with T2DM who had poor glycemic control usually require frequent follow-ups and higher numbers of clinical and laboratory examinations compared with those with good glycemic control. The higher frequency of follow-up and consultation may contribute to the higher PACIC-M score.

### Strengths and limitations of the study

There was scarce evidence on patients’ perception of diabetes care among people with T2DM in primary healthcare clinics in Malaysia. To the best of our knowledge, this study is the first to report the assessment of diabetes care among patients with T2DM in Kelantan using the PACIC-M questionnaire with a relatively large sample. The study findings are representative of the total diabetes population in Kelantan due to the adequate sample from all districts. Although a self-administered PACIC-M questionnaire was originally developed, a structured interview-guided technique was used to obtain the information. This approach has allowed people with lower health literacy skills to participate, hence high response rate. Interviewer bias was minimized as only one interviewer was trained and involved in data collection.

In this study, data regarding the number of available medical officers in a clinic were extracted, rather than the exact number of medical officers seen by the patients in a particular clinic. Thus, the interpretation of the number of doctors seen by a patient would be inaccurate. Moreover, the researcher was unable to gather data regarding the frequency of visits of a patient to an FMS per year. People with T2DM who were managed by FMS could score differently from those who were managed by a medical officer only, as patients who consulted FMS usually had more complicated diabetes care and required more detailed management and counseling. Nonetheless, information regarding the numbers of diabetes educators was gathered, but the people with T2DM were not asked whether they received any form of diabetes education, which may affect how they perceive the quality of care.

### Implication and future research

The current study provides knowledge about the difference in perceived quality of care received by people with T2DM in different healthcare clinic settings and factors that influenced it. However, to achieve a better understanding on the effects of the numbers of medical officers and FMS in increasing the perceived quality of care, a further cohort study following an increased number of resident FMS in clinics can be conducted to evaluate the causal effects of such number of FMS toward the perceived quality of care. Further research to compare the perceived quality of care among patients with chronic conditions, including diabetes, managed by FMS and medical officers should also be conducted. The components of high-quality chronic disease care, including the continuity of care (whether patients are seeing the same doctor), comprehensive care (whether patients are seeing PHCT, such as a diabetes educator), and coordination of care (defaulter tracing, referrals, or home visits), should be assessed to evaluate the implementation of FDC in Kelantan in particular and Malaysia as a whole. Apart from that, an evaluation of the implementation of FDC in Kelantan and the health outcome thereof could be conducted to determine that FDC clinics are able to produce better health outcome in long term.

## Conclusions

The quality of care was perceived equally in both FDC and non-FDC clinics, and it was associated with a lower number of medical officers and good satisfaction in doctor–patient interaction. Although people with T2DM perceived similar quality of care regardless of the type of healthcare clinics they visited, restructuring the primary healthcare into FDC, enhancing FDC implementation, and promoting “One Family One Doctor” are good initiatives as patients experience better continuity of care and doctor–patient interaction satisfaction. However, the number of family physicians in primary healthcare clinics should be increased to deliver better, coordinated diabetes care.

## Data Availability

The datasets generated and/or analyzed during the current study are not publicly available due to confidentiality issues but are available from the corresponding author on reasonable request.

## Abbreviations

CCM: Chronic care model
FDC: Family Doctor Concept
FMS: family medicine specialist
HbA1c: glycated hemoglobin A1c
PACIC-M: Patient Assessment of Chronic Illness Care (Malay version) questionnaire
SKIP-11: *Skala Kepuasan Interaksi Perubatan-11* questionnaire
T2DM: type 2 diabetes mellitus
PHCT: primary healthcare team
